# Trends and Challenges in Noninvasive Hemodynamic Monitoring of Neonates Following Cardiac Surgery: A Narrative Review

**DOI:** 10.3390/life15101621

**Published:** 2025-10-17

**Authors:** Carmina Nedelcu, Nicolae Sebastian Ionescu, Ana Mihaela Bizubac, Cristina Filip, Catalin Cirstoveanu

**Affiliations:** 1 Doctoral School of “Carol Davila” University of Medicine and Pharmacy, 020021 Bucharest, Romania; 2 Department of Neonatal Intensive Care, “Carol Davila” University of Medicine and Pharmacy, 020021 Bucharest, Romania; mihaela.bizubac@drd.umfcd.ro (A.M.B.); catalin.cirstoveanu@umfcd.ro (C.C.); 3 Neonatal Intensive Care Unit, “M.S. Curie” Children’s Hospital, Constantin Brâncoveanu Boulevard, No. 20, 4th District, 041451 Bucharest, Romania; filipcristina06@yahoo.com; 4 Department of Pediatric Surgery and Orthopedics, “Carol Davila” University of Medicine and Pharmacy, 020021 Bucharest, Romania; 5 Romanian Academy of Medical Sciences, 030167 Bucharest, Romania; 6 Romanian Academy of Scientists, 030167 Bucharest, Romania; 7 Department of Pediatrics, “Carol Davila” University of Medicine and Pharmacy, 020021 Bucharest, Romania

**Keywords:** hemodynamic monitoring, congenital heart disease, cardiac surgery, neonatal intensive care

## Abstract

Hemodynamic monitoring is essential in the postoperative management of neonates undergoing cardiac surgery, enabling early identification of circulatory failure and its underlying cause, optimization of oxygen delivery to tissues, and evaluation of treatment response. Despite its significant role, there is still no consensus and there remains substantial heterogeneity in bedside hemodynamic monitoring practices. Pediatric intensivists typically rely on macro- and microcirculatory indicators, including arterial blood pressure, urine output, capillary refill time, mixed venous oxygen saturation, lactate concentration, and serial echocardiographic evaluations. However, most of these are indirect hemodynamic indicators and provide only intermittent snapshots of the hemodynamic status, which can be very fluctuating following cardiac surgery. Technological advancements have driven a shift toward continuous, noninvasive monitoring techniques, such as near-infrared spectroscopy (NIRS), electrical biosensing technology, and microcirculatory assessment tools. Real-time, simultaneous tracking of multiple physiological variables through a multimodal hemodynamic monitoring protocol facilitates the understanding of systemic and regional perfusion and oxygenation. This narrative review aims to summarize current techniques and innovations in neonatal hemodynamic monitoring following cardiac surgery, combining clinical evaluation with both intermittent and continuous noninvasive techniques.

## 1. Introduction

Hemodynamic monitoring is one of the most challenging yet vital components in the care of critically ill neonates and infants following cardiac surgery due to the heterogeneity of neonatal physiology, comorbidities, and the intricate relationship between systemic and regional perfusion. Hemodynamic evaluation is based on cardiac output (CO) and systemic vascular resistance (SVR), which together determine systemic blood flow and tissue perfusion. Intermittent clinical evaluation and continuous measurement of standard parameters, such as heart rate, arterial oxygen saturation, and blood pressure, are often inadequate for comprehensive hemodynamic evaluation given the dynamic nature of the cardiovascular system after surgery [1].

Low cardiac output syndrome (LCOS) is characterized by insufficient delivery of oxygenated blood to meet the metabolic demands of tissues, leading to tissue hypoxia and metabolic acidosis [2]. It is the most common postoperative complication in children with congenital heart disease (CHD), with an incidence ranging between 25 and 60% and typically manifesting within the first 6 to 18 h following surgery [3]. LCOS is caused by multiple factors, such as the underlying heart disease and cardiac function, the use and duration of cardiopulmonary bypass, intraoperative factors, residual lesions, arrhythmias, and the patient’s baseline status.

A standardized definition of LCOS is lacking but commonly used definitions share criteria such as signs of poor tissue perfusion, metabolic acidosis, and reduced cardiac output. Wernovsky et al. define LCOS based on a cardiac index less than 2 L/min/m^2^ [4]. Hoffman et al. describe LCOS as a clinical syndrome with tachycardia, oliguria, cold extremities, and/or cardiac arrest, with or without an arterial–mixed venous oxygen saturation difference ≥ 30%, or metabolic acidosis (base deficit > 4 mmol/L or lactate concentration > 2 mg/dL) on two consecutive blood gas measurements [5]. The Pediatric Cardiac Critical Care Consortium (PC4) includes the vasoactive-inotropic score (VIS) > 15, a tripling of VIS to >10 within 48 h, arterio-venous oxygen difference > 40% with hemoglobin > 8 g/dL, and clinical diagnosis of LCOS [6]. A systematic review analyzing definitions of LCOS in infants, children, and adults found that in pediatric cases, metabolic acidosis appeared in 64% of definitions, the need for inotropic support in 61%, and clinical manifestations such as oliguria, tachycardia, cold extremities, and altered mental status in 59% [7]. Other less frequent indicators included increased arterial–venous oxygen saturation difference (35%), cardiac arrest (28%), hypotension (25%), impaired cardiac pump function (20%), and the requirement for mechanical circulatory support (20%).

The Royal College of Anesthetists recommends for cardiac output monitoring to be readily available for pediatric patients undergoing cardiac surgery [8]. However, the guideline does not recommend a specific method, highlighting the lack of a gold standard in pediatric hemodynamic monitoring, which leads to variability between institutions regarding the postoperative hemodynamic surveillance protocol [9].

Cardiac output monitoring techniques can be categorized by their invasiveness. Invasive methods are the gold standard in adult patients, but they require advanced technical expertise, carry risks of procedural complications, require sedation, and are associated with higher costs. These disadvantages limit their routine use in neonates and small infants.

Cardiac output measurement reflects the macrocirculatory status, but it does not necessarily reflect organ perfusion. Therefore, a comprehensive hemodynamic monitoring protocol should include both systemic and regional assessments, such as near-infrared spectroscopy (NIRS) and microcirculatory evaluation tools, including laser Doppler flowmetry, orthogonal polarization spectral imaging, sidestream dark field imaging, and incident dark field imaging.

This narrative review aims to explore the current methods used for hemodynamic monitoring in neonates and small infants with congenital heart disease following cardiac surgery, as well as emerging technologies, focusing on noninvasive and minimally invasive continuous monitoring devices, their advantages and limitations in clinical practice. The main objective is to identify the most suitable methods for this population, regarding accuracy, safety and ease of use, that can be incorporated in a protocol for global hemodynamic monitoring at the patient’s bedside in a Post-Cardiac Neonatal Intensive Care Unit.

## 2. Materials and Methods

We conducted a systematic database search in PubMed and Embase within the last 10 years (2015–2025). One reviewer performed the search using the following terms: “neonate” OR “newborn” OR “infant” AND “congenital heart disease” OR “heart disease” OR “CHD” AND “cardiac surgery” OR “cardiac procedure” OR “cardiac intervention” AND “hemodynamic” OR “hemodynamics”. Additional articles were identified by manual search. Titles and abstracts were screened for relevance and duplications. Articles with full texts in other language than English were excluded. More details on the database search and on the selection of studies can be found in Appendix A (see Table A1 and Table A2).

## 3. Indirect Hemodynamic Monitoring

Systemic blood flow and oxygenation are most frequently assessed indirectly using clinical and biochemical markers, which are essential in bedside monitoring, but their accuracy and predictive value in diagnosing circulatory failure is limited [1]. Low cardiac output state is accurately clinically recognized in only 25% of cases in pediatric intensive care units [10].

### 3.1. Blood Pressure

Blood pressure remains the most employed clinical parameter for hemodynamic evaluation in the NICU, despite its limited accuracy in reflecting systemic perfusion. Following cardiac surgery, patients already have central arterial and venous catheters in place for therapeutic management, facilitating invasive monitoring of arterial and central venous pressures.

A universally accepted definition of neonatal hypotension is still lacking [11]. Some definitions rely on thresholds below the 10th or 5th percentile of normative blood pressure, adjusted for gestational age and postnatal age. Others define hypotension as a mean arterial pressure below 30 mmHg, based on the assumption that cerebral perfusion becomes pressure-dependent at this level. The main goal of hypotension prevention and management is to ensure adequate perfusion to end-organs. However, both hypotension and hypertension, as well as fluctuating blood pressure, are associated with adverse clinical outcomes.

The most recent consensus statement from the Cardiovascular Dynamics Section of the European Society of Pediatric and Neonatal Intensive Care (ESPNIC) advises against relying solely on blood pressure as a therapeutic target in hemodynamically unstable children, instead advocating for a comprehensive assessment that integrates blood pressure with a range of clinical and paraclinical parameters [12]. Simultaneous assessment of blood pressure and cardiac output allows a pathophysiology-oriented management of the circulatory failure [13]. Furthermore, a normal blood pressure does not imply a normal cardiac output. A compensatory increase in systemic vascular resistance can keep the blood pressure normal despite a low cardiac output state.

**Figure 1:**
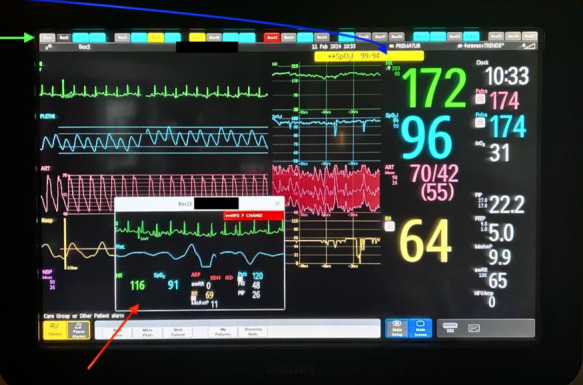
Central monitoring showing heart rate, oxygen saturation, arterial blood pressure, respiratory rate and their trend during the last 9 h, along with ventilator parameters. The blue arrow shows that there is an active alarm for this patient due to over-the-limit oxygen saturation. The green arrow shows the alarm status for all the other patients. The red arrow is a “pop-up” alarm, showing a sudden change in respiratory rate in another patient. Image from the Neonatal Intensive Care Unit at the “Maria Sklodowska Curie” Emergency Clinical Hospital for Children.

Trend monitoring of systolic, diastolic, and mean arterial pressures, along with pulse pressure analysis, can offer insight into hemodynamics (see Figure 1). Pulse pressure, defined as the difference between systolic and diastolic pressures, serves as a valuable hemodynamic marker. A widened pulse pressure accompanied by low diastolic pressure is often caused by left-to-right shunts (e.g., patent ductus arteriosus or Blalock–Taussig shunt), aortic regurgitation, or severe anemia. A narrow pulse pressure can be seen in low cardiac output states or during extracorporeal membrane oxygenation (ECMO) [14].

The morphology of the arterial pressure waveform depends on the systolic and diastolic pressures and is influenced by cardiac output, systemic vascular resistance, arterial compliance, and blood viscosity. Alterations of the arterial pressure waveform may indicate left ventricular dysfunction, cardiac tamponade, or aortic insufficiency [14].

Central venous pressure (CVP), measured at the junction of the superior vena cava and right atrium, remains a vital part of the hemodynamic monitoring post-cardiopulmonary bypass, despite its limited utility in assessing fluid responsiveness [15,16]. CVP provides an estimate of right atrial pressure, reflecting preload and right ventricular function. While low CVP values are often caused by hypovolemia, elevated values may suggest fluid overload or right ventricular failure leading to venous congestion. Current clinical guidelines emphasize the utility of CVP monitoring and recommend maintaining CVP at the lowest feasible level [15]. The most recent ESPNIC consensus on hemodynamic monitoring states that CVP should not be employed as a standalone parameter to guide fluid therapy, and that its interpretation requires a thorough understanding of its inherent limitations [12].

### 3.2. Heart Rate

Electrocardiographic (ECG) monitoring following cardiac surgery allows the assessment of heart rate, pacemaker function, and detection of arrhythmias, myocardial ischemia, and acute ST-segment changes, particularly after coronary artery reimplantation during procedures such as arterial switch operation [14].

In neonates, cardiac output is predominantly dependent on heart rate, with less variability of the stroke volume. Tachycardia is often a compensatory mechanism for reduced systemic perfusion, whereas a stable heart rate indicates hemodynamic stability. However, elevated heart rates can effectively maintain cardiac output only if end-diastolic volume remains sufficient. Excessively high heart rates may impair myocardial perfusion and contractility by reducing ventricular filling time.

Heart rate is influenced by various external factors, such as temperature, pain, and pharmacologic agents such as inotropes. As a result, sporadic heart rate assessments are unreliable hemodynamic indicators. On the other hand, changes in heart rate trends can signal alterations in cardiac output, which makes continuous monitoring more informative.

Heart rate variability (HRV), defined as the variation in time intervals between consecutive heartbeats (R-R intervals), provides a noninvasive measure of autonomic nervous system function through spectrum analysis. Reduced HRV is associated with impaired autonomic regulation and elevated risk of hemodynamic instability and has been observed in patients with congenital heart defects before and after surgeries involving cardiopulmonary bypass [17,18].

### 3.3. Urine Output

Kidneys are highly sensitive to low cardiac output, which makes hourly urine output monitoring a key parameter following cardiac surgery. Furthermore, changes in urine output can indirectly reflect the effectiveness of therapeutic interventions, such as fluid resuscitation or administration of inotropic and vasoactive agents.

According to the KDIGO guidelines, acute kidney injury (AKI) is diagnosed based on elevated serum creatinine levels and/or diminished urine output [19]. Urine output is regarded as a sensitive and early marker of renal dysfunction [20]. Postoperative urine output monitoring facilitates the early detection of AKI, which has been reported in approximately 53.8% of neonates undergoing congenital heart surgery [21]. Several perioperative factors contribute to cardiac surgery-associated AKI development, including cardiopulmonary bypass, aortic cross-clamping, low cardiac output syndrome, administration of vasoactive agents and albumin, transfusions, inflammation, and clotting abnormalities [22].

### 3.4. Capillary Refill Time

Capillary refill time (CRT) is a clinical tool for assessing microcirculatory status in pediatric patients. Even though it does not provide direct information about cardiac output, CRT is frequently used at the bedside due to its simplicity, noninvasiveness, rapidness, low cost, and good interobserver reliability [23]. CRT has been validated as a rapid response marker during septic shock resuscitation and is considered useful for evaluating the effectiveness of fluid therapy or vasoactive medication [23]. A rapid return of CRT to baseline suggests preserved hemodynamic coherence between macro- and microcirculations [24].

### 3.5. Central–Peripheral Temperature Difference

Peripheral vasoconstriction secondary to hypoperfusion reduces peripheral skin temperature, thereby increasing the temperature gradient between central (e.g., thorax or abdomen) and peripheral (e.g., hands or feet) regions. Under normal conditions, the central–peripheral temperature difference (CPTd) should be within a 3–4 °C range; however, it can be influenced by ambient temperature and vasoactive drugs [25].

Skin temperature can be assessed qualitatively through palpation or quantitatively through skin thermometry or infrared thermography (IRT). IRT, a noninvasive imaging modality, offers objective and reproducible measurements, which makes it suitable for evaluating peripheral perfusion and microcirculation [26]. Multiple studies have demonstrated a positive correlation between CPTd and systemic vascular resistance, as well as inverse relationships with cardiac output, stroke index, and urine output [25,27]. Nonetheless, other studies did not find any statistically significant associations [28,29].

A combined clinical–biological scoring system for low cardiac output syndrome (LCOS), incorporating qualitative CRT and quantitative toe temperature assessments, has been associated with increased postoperative morbidity [30]. Additionally, a central–peripheral temperature difference > 5 °C has been used as a marker in several studies, showing associations with decreased cardiac output and elevated peripheral vascular resistance [25,31].

### 3.6. Lactate

Serum lactate concentration is widely used to monitor postoperative LCOS. According to a survey study, 99% of pediatric intensivists include it in their assessments [32]. Both Ulate et al. [30] and Aslan et al. [33] integrated lactate measurement into their LCOS scoring systems as a marker of tissue perfusion. Despite its widespread use, lactate is not a sensitive early biomarker of reduced cardiac output. Lactate levels typically increase only after oxygen delivery has fallen below a critical threshold and cellular oxygen extraction becomes insufficient to meet the metabolic demand [1]. Moreover, lactate may accumulate locally and remain undetected until perfusion is restored, leading to lactate mobilization and increase.

Elevated lactate concentrations have been associated with increased mortality and a higher risk for requiring ECMO post-cardiac surgery [34]. Strategies aimed at reducing lactate levels have been linked to improved clinical outcomes, reduced organ dysfunction and lower mortality rates in various shock states [35]. However, aggressive efforts to normalize lactate concentrations may lead to fluid overload, especially in the absence of other signs of tissue hypoperfusion [36,37].

### 3.7. Venous Oxygen Saturation

Mixed venous oxygen saturation (SvO_2_) is a marker of global tissue oxygenation, reflecting the residual oxygen reserve following tissue oxygen extraction. SvO_2_ is influenced by multiple factors, including arterial oxygen concentration, oxygen consumption, cardiac output, and hemoglobin concentration.

SvO_2_ is measured using a pulmonary artery catheter, but this is not feasible in neonates. As an alternative, central venous oxygen saturation (ScvO_2_) is commonly utilized. Although SvO_2_ and ScvO_2_ are not entirely interchangeable, changes in ScvO_2_ reflect fluctuations in SvO_2_. Monitoring venous oxygen saturation trends is more informative than focusing on absolute values.

In pediatric patients undergoing cardiac surgery, ScvO_2_ levels < 68% associated with lactate concentrations > 3 mmol/L during cardiopulmonary bypass have been correlated with increased morbidity and mortality [38]. Therapeutic strategies aimed at optimizing ScvO_2_ have been associated with improved clinical outcomes, reduced organ dysfunction, decreased need for vasoactive drugs, decreased mechanical ventilation duration, and enhanced survival rates [39].

Another parameter serving as a surrogate for SvO_2_ is the veno-arterial carbon dioxide (v-aPCO_2_) gradient. A gradient > 6 mmHg is suggestive of impaired tissue perfusion [40]. In line with Fick’s principle, both oxygen consumption and carbon dioxide production are directly proportional to cardiac output. Recent research identified the ratio of the veno-arterial carbon dioxide pressure difference to the arterial–venous oxygen content difference [P(v-a)CO_2_/C(a-v)O_2_] as a potential marker of tissue hypoxia secondary to circulatory failure [41]. A high ratio is caused by increased CO_2_ production and/or reduced O_2_ extraction.

### 3.8. Vasoactive–Inotropic Score

The vasoactive–inotropic score (VIS) quantifies the type and dosage of pharmacologic agents required to sustain adequate perfusion and hemodynamic stability. VIS serves as an indirect measure of clinical severity and of circulatory support needed [42]. Gaies et al. applied VIS to neonates and small infants after cardiac surgery, defining high VIS as ≥20 during the first 24 h or ≥15 during the 24–48 h postoperative period [43]. Elevated VIS was associated with adverse composite outcomes, including mortality, need for mechanical circulatory support, renal replacement therapy, cardiac arrest, and central nervous system injury.

Miletic et al. introduced the vasoactive–ventilation–renal (VVR) score, which integrates cardiovascular, respiratory, and renal parameters [44]. The VVR score has shown utility in predicting postoperative outcomes and mortality in pediatric cardiac surgery patients [45]. In a multicenter study focusing exclusively on neonates, a VVR score ≥ 35 at 48 h post-surgery was more predictive of prolonged mechanical ventilation than VIS, the ventilation index (VI), or lactate levels [46]. Another study identified a peak VVR ≥ 46.5 within the first 72 h after surgery as a marker of increased risk for adverse outcomes in neonates with congenital heart disease [47]. A summary of the calculating formulas for the vasoactive–inotropic score (VIS), ventilation index (VI) and vasoactive–ventilation–renal score (VVR) can be found in Table 1.

### 3.9. Low Cardiac Output Syndrome Score

The low cardiac output syndrome score (LCOSS) incorporates clinical observation and therapeutic interventions, but it does not provide a direct measurement of cardiac output. The major advantages are its simplicity and bedside applicability. Ulate et al. developed a scoring system for infants with congenital heart disease undergoing surgery. The LCOSS assigns one point for each of the following: tachycardia, oliguria, decreased toe temperature, fluid requirement, decreased cerebral oxygen saturation measured by NIRS, elevated lactate levels, and inotropic/vasoactive support [30]. The LCOSS has demonstrated significant correlation with the severity and duration of low cardiac output state, morbidity, and the length of intensive care unit stay. A similar scoring method was later used by Aslan et al., who substituted toe temperature with prolonged capillary refill time in their evaluation of pediatric post-cardiac surgery patients (see Table 1) [33].

## 4. Direct Hemodynamic Monitoring

Many methods for direct hemodynamic assessment are challenging to implement in the monitoring of newborns due to size restraints and presence of intra-/extracardiac shunts.

### 4.1. Echocardiography

Targeted neonatal echocardiography (TNE) is routinely used in the NICU to assess the hemodynamics of critically ill newborns. Beyond hemodynamic evaluation, echocardiography allows a comprehensive assessment of cardiac morphology, intracardiac pressures, volumes, systolic and diastolic functions [49]. When used in association with clinical examination and other indirect hemodynamic markers, bedside echocardiography provides immediate, noninvasive diagnostic and functional information. TNE can lead to changes in clinical management in approximately 30–60% of patients with hemodynamic instability [50]. However, accurate image acquisition and interpretation require training of the neonatologist, as well as a close collaboration with pediatric cardiologists [51].

**Figure 2:**
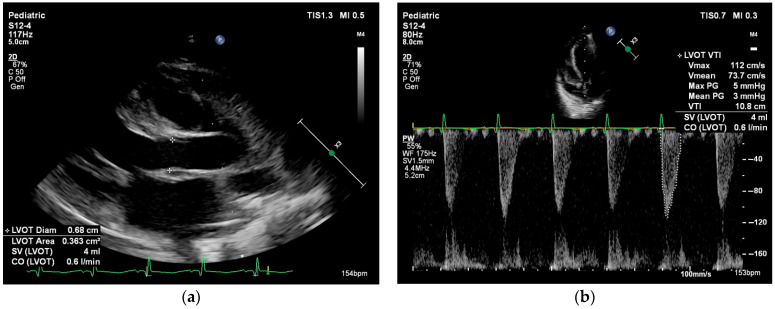
Cardiac output measurement performed in the parasternal long axis view in a neonate with myocarditis. Cardiac output = stroke volume × heart rate. Stroke volume = velocity time integral × cross-sectional area. (**a**) Left ventricle outflow tract (LVOT) cross-sectional area = π × (LVOT diameter/2)^2^. (**b**) Velocity time integral is measured using pulsed wave Doppler at the level of the LVOT.

Systemic blood flow (left ventricular output, LVO) and pulmonary blood flow (right ventricular output, RVO) can be estimated by calculating the product of the Doppler flow velocity (velocity time integral, VTI), the cross-sectional area (CSA) of the left or right ventricular outflow tracts, and heart rate. In the presence of intra- or extracardiac shunts, LVO and RVO are not interchangeable. There are normative data for neonatal VTI and cardiac index, but these measurements are highly dependent on correct ultrasound probe positioning and accurate estimation of the outflow tract diameter (see Figure 2) [52].

In neonates without shunting, echocardiographic measurement of cardiac output is generally reliable, with values ranging between 150 and 300 mL/kg/min [53,54]. Echocardiography is the most widely used direct method for hemodynamic evaluation, even though its precision is limited compared to invasive methods. For instance, echocardiography shows a precision error of around 30% when compared to gold standard dilution techniques, which falls within the clinically accepted range [54,55]. Similar levels of variability have been noted in comparison to cardiac MRI [56]. Anatomical variations can further reduce the accuracy of echocardiographic cardiac output measurements in patients with congenital heart disease [57]. Therefore, serial echocardiographic evaluations are often more informative than single-point assessments, allowing a more dynamic monitoring of the patient’s response to treatment and evolving hemodynamic status.

Despite its known limitations, including relatively high intra- and interobserver variability (coefficient of variation ranging from 2.1% to 22% and 3.1% to 22%, respectively) [58], echocardiography remains the most widely adopted modality in neonatal and pediatric intensive care. Therefore, it is also the reference method for validation of emerging hemodynamic monitoring techniques.

The most recent ESPNIC consensus for hemodynamic monitoring in critically ill children recommends the echocardiographic measurement of cardiac output in clinical practice [12]. Despite being the most accurate for cardiac output measurement, transpulmonary dilution methods are not suitable in clinical practice for monitoring rapid and frequent hemodynamic changes due to their invasiveness, technical difficulties and lack of expertise.

### 4.2. Cardiac Magnetic Resonance Imaging

Cardiac magnetic resonance imaging (CMRI) is considered the noninvasive gold standard method for quantifying cardiac output [59]. It allows detailed evaluation of ventricular volumes and function, myocardial tissue edema, fibrosis, and scarring, thus facilitating the diagnosis of heart failure, myocarditis and congenital heart disease (e.g., borderline hypoplastic left ventricle) [59]. In patients without intracardiac shunting, CMRI can internally validate cardiac output measurements by comparing volumetric or flow-based data from both ventricles [60].

CMRI is more accurate and comprehensive than transthoracic echocardiography. However, it is not routinely used in acute settings for rapid assessment of hemodynamic changes due to a longer acquisition time, need for patient sedation and because it requires interpretation from an experienced cardiac radiologist.

### 4.3. Transcutaneous Doppler

Cardiac output can be estimated noninvasively using transcutaneous Doppler (TCD), such as the Ultrasonic Cardiac Output Monitor (USCOM). The device allows the measurement of blood flow velocity, typically at the level of the ascending aorta or pulmonary artery. Thus, it enables rapid bedside assessment of cardiac output without requiring extensive training in echocardiography.

Unlike transthoracic echocardiography, the cross-sectional areas of the left and right ventricular outflow tracts are not measured through direct visualization but approximated through algorithms that consider the patient’s age, height, and weight. Consequently, TCD exhibits lower accuracy due to limited measurement precision. It has poor agreement to other cardiac output monitoring techniques, including TTE and thermodilution, with a bias ranging from 0% to 21% and an error margin between 43 and 65%, respectively [54]. Multiple investigations have concluded that USCOM is not suitable for routine use in pediatric cardiac populations [61].

Cerebral hemodynamics can be monitored using transcranial Doppler techniques or serial transfontanellar Doppler ultrasound assessments, particularly during the perioperative period. Clinicians can assess cerebral autoregulatory function in relation to systemic blood pressure by measurement of cerebral blood flow velocities (CBFVs) along with mean arterial pressure and near-infrared spectroscopy (NIRS) [62].

NeoDoppler is a device specifically developed for continuous transfontanellar Doppler monitoring in neonates and infants. In a small, single-center study by Olsen et al., this technology was found to be a potentially valuable tool for real-time cerebral monitoring during transcatheter interventions in infants with CHD [63]. The authors concluded that NeoDoppler may aid both anesthesiologists and interventional cardiologists by identifying early signs of neurologic or hemodynamic instability. More recently, the same group demonstrated that NeoDoppler could continuously track CBFV fluctuations throughout cardiopulmonary bypass in infants undergoing various cardiac surgical procedures, and that it could detect hemodynamic changes more rapidly than NIRS [62].

### 4.4. Near-Infrared Spectroscopy

**Figure 3:**
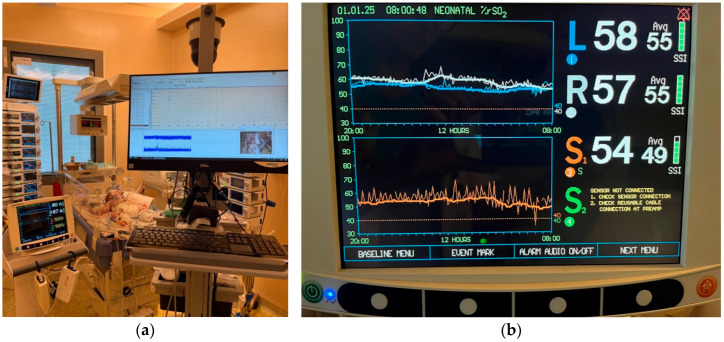
Neuromonitoring of infants following cardiac surgery in the NICU, consisting of (**a**) amplitude integrated electroencephalography (aEEG) monitoring before the surgery and in the first 24 h postoperative, (**a**,**b**) continuous NIRS monitoring perioperative (initiated before the intervention and continued until extubation), as well as transfontanellar Doppler before and after intervention (measuring blood flow velocities and resistivity index).

Near-infrared spectroscopy (NIRS) has been increasingly used as a hemodynamic monitoring tool in many Post-Cardiac Pediatric Intensive Care Units (see Figure 3). The ESPNIC committee on hemodynamic monitoring recommends the employment of NIRS during the peri-operative period after surgery for congenital heart defects [12]. This noninvasive method allows real-time assessment of regional tissue oxygenation and perfusion.

NIRS was proved efficient in identifying low cardiac output syndrome (LCOS), leading to its routine use after cardiopulmonary bypass [64]. Cerebral oxygen saturation (ScO_2_) acts as a marker of cardiac output if ventilation, oxygenation, and hemoglobin levels remain stable [65].

Systemic parameters such as arterial blood pressure, heart rate, arterial oxygen saturation, and partial pressure of carbon dioxide can influence cerebral perfusion, but ScO_2_ reflects the dynamic relationship between oxygen delivery to the brain and cerebral metabolic demand. NIRS values are very responsive to physiological changes, signaling alterations in cerebral oxygenation more rapidly than traditional vital sign monitoring. This makes NIRS effective for early detection of hemodynamic disturbances, guiding timely therapeutic interventions and predicting short- and long-term outcomes. Aly et al. reported that a cerebral oxygen saturation threshold of <58%, when combined with a lactate concentration > 7.4 mmol/L at 24 h post-neonatal cardiac surgery, served as a reliable predictor of survival and neurodevelopmental outcomes [66]. Similarly, in a large-scale investigation, Hoffman et al. demonstrated that NIRS measurements at 6 h after palliative surgery for hypoplastic left heart syndrome were strong predictors of early mortality and the need for extracorporeal membrane oxygenation [67].

Nonetheless, clinicians need to consider several limitations of NIRS, such as intra- and inter-patient variability, device and sensor variability, sensor position, signal contamination due to head position, phototherapy or day-to-day interventions (handling of patient, endotracheal suctioning, etc.), as well as the high cost.

Average ScO_2_ values range between approximately 65 and 70% in both term and preterm neonates [68,69], in comparison to 46–57% in patients with cyanotic congenital heart disease [12]. Many studies have investigated the normal values of tissue-specific oxygen saturation in the neonatal population, but most of them were small, single-center trials and used different NIRS devices and sensors. For this reason, there is still no universally accepted lower threshold guiding intervention and no consensus on the clinical utility of a decline of cerebral oxygen saturation < 40% or a change in baseline > 20% [12]. Clinical use of NIRS monitoring is based on the determination of each patient’s baseline ScO_2_ under stable conditions. This individualized approach allows clinicians to detect disturbances post-cardiac surgery by comparing it against the patient’s own “normal” status.

### 4.5. Thoracic Electrical Biosensing Technology

Electrical biosensing technology (EBT) enables noninvasive, real-time, continuous monitoring of hemodynamic parameters directly at the patient’s bedside. Low-amplitude, high-frequency electrical current is delivered through surface electrodes and is predominantly distributed to the vascular system due to the high conductivity of blood. Variations in electrical impedance over time reflect stroke volume, allowing for the calculation of cardiac output when combined with heart rate.

EBT can be categorized into two main types: bioimpedance (BI) and bioreactance (BR), which differ in terms of signal processing techniques, electrode placement, and the algorithms employed to calculate stroke volume.

**Figure 4:**
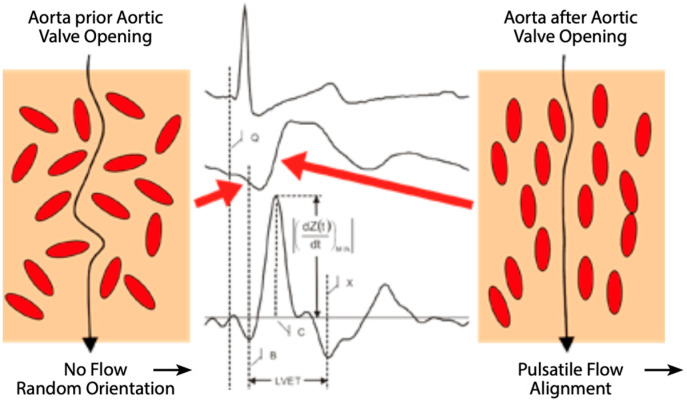
Random arrangement and orientation of red blood cells during diastole vs. parallel alignment with aortic flow during systole. This results in decreased impedance and increased conductivity. The rate of change in conductivity before and after aortic valve opening leads to the peak aortic acceleration of blood, left ventricular ejection time, and ultimately blood flow velocity, which is used to calculate stroke volume and cardiac output. Figure from ICON brochure, Osypka Medical GmbH.

**Figure 5:**
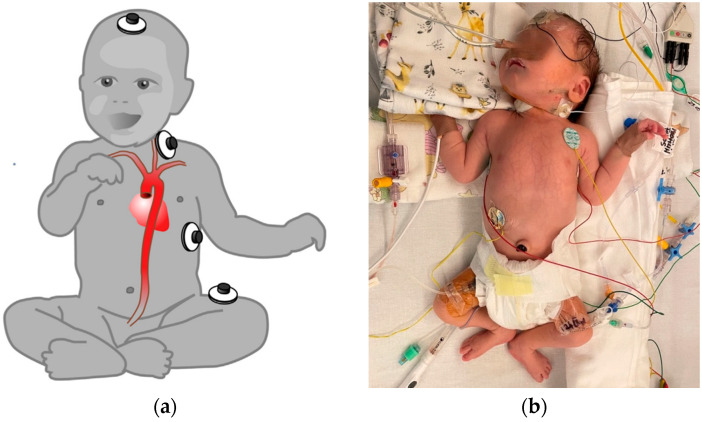
(**a**) Sensor placement for small children and neonates according to recommendations from the manufacturer Osypka Medical GmbH. (**b**) Sensor placement illustrated in a patient in the post-cardiac surgery period in the Neonatal Intensive Care Unit.

Electrical cardiometry (EC), a type of thoracic bioimpedance monitoring technology, allows the evaluation of cardiac output and other related hemodynamic variables. The ICON monitor (Osypka Medical Inc., Berlin, Germany) is FDA-approved for patients of all ages, including neonates (see Figure 4 and Figure 5). It is a compact, user-friendly, and cost-effective tool for noninvasive hemodynamic assessment.

Multiple studies have evaluated EC in neonates and infants with congenital heart disease, reporting statistically significant variations in cardiac index and left ventricular ejection fraction among heart failure subgroups [70]. Moreover, EC has shown strong agreement with transthoracic echocardiography regarding stroke volume, stroke volume variation (SVV), and the inferior vena cava distensibility index (dIVC) [71].

Grollmuss et al. compared stroke volume measurements obtained through EC and TTE in preterm infants with low and very low birth weights [72], while Noori et al. examined left ventricular output in preterm neonates with and without patent ductus arteriosus, finding good agreement between the two modalities [73]. Additionally, EC has shown low bias when compared with TTE in infants postoperatively [74]. However, most of the studies investigating electrical cardiometry in neonates are small and single-center, which limits statistical power and reduces the generalizability of the findings. Larger, multicenter studies are needed to validate these results.

In a more recent investigation, Ibrahim et al. examined the relationship between cardiac output measurements by EC and TTE, and clinical status in infants following cardiac surgery [75]. ICON-derived parameters such as thoracic fluid content (TFC) correlated with fluid balance and cardiopulmonary bypass duration. EC and echocardiography have also shown significant correlations regarding contractility indices (e.g., TAPSE, cardiac index) and fluid status (e.g., IVC collapsibility and TFC).

Cardiac output estimated via EC has also shown good correlation with cardiac magnetic resonance imaging (CMRI) in pediatric patients with congenital heart defects, although EC tends to slightly overestimate CO [60].

**Figure 6:**
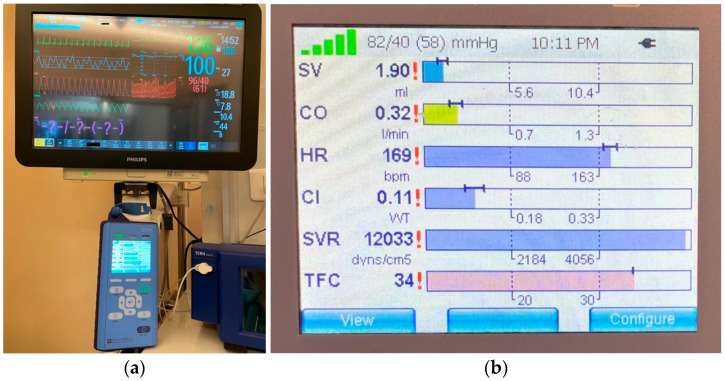
(**a**) Hemodynamic monitoring through electrical cardiometry (ICON) in association with standard hemodynamic monitoring in the Neonatal Intensive Care Unit. (**b**) The major hemodynamic parameters that we focus on in the Post-Cardiac NICU (stroke volume, heart rate, cardiac output, cardiac index, systemic vascular resistance, thoracic fluid content).

Thoracic fluid content is another hemodynamic parameter which can be evaluated through electrical cardiometry (see Figure 6). It has proven useful in a variety of clinical scenarios, such as predicting outcomes in critically ill children [76], evaluating fluid responsiveness in children with shock [77], detecting pulmonary congestion [78], monitoring fluid overload during hemodialysis [79], and distinguishing between hemodynamically significant and closing/restrictive patent ductus arteriosus (PDA) [80].

**Figure 7:**
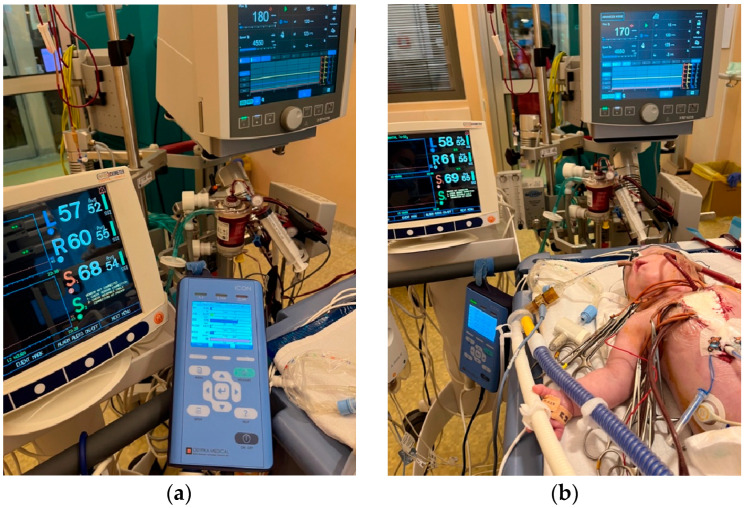
(**a**) Hemodynamic monitoring (electrical cardiometry, NIRS) of a preterm neonate with pulmonary valve atresia on ECMO support and CRRT following the surgical procedure (modified Blalock-Taussig shunt). (**b**) The image from the NICU at the “Maria Sklodowska Curie” Emergency Clinical Hospital for Children shows consistency between the cardiac output provided by veno-arterial ECMO support and the cardiac output measured by ICON.

Limitations of electrical cardiometry include sensor placement and interaction with other sensors placed nearby (ECG, NIRS), signal quality, motion artifact, interaction with high-frequency oscillatory ventilation, heart rate variability or arrhythmias, as well as limited validation in specific types of congenital heart defects (see Figure 7). Shunts may lead to underestimation or overestimation of systemic blood flow depending on the magnitude and direction of the shunt.

The Special Interest Group on Noninvasive Cardiac Output Monitoring (NICOM) of the European Society for Pediatric Research (ESPR) has recently issued guidance on EBT use in neonates. The consensus statement indicates that thoracic EBT is not currently reliable for precise measurement of cardiac output in neonates and should not replace TTE, which remains essential for assessing cardiac anatomy and function, as well as for confirming EBT-derived findings. Nonetheless, EBT can be clinically useful for monitoring changes from the individual baseline [81].

### 4.6. Microcirculatory Monitoring Devices

The main objective of hemodynamic resuscitation is to improve microvascular perfusion to ensure sufficient oxygen delivery to tissues and meet the metabolic requirements. Even after improving the macrocirculatory parameters, such as blood pressure and cardiac output, it remains uncertain whether adequate improvement in microcirculation and tissue oxygenation has occurred. The “loss of hemodynamic coherence” describes a clinical condition characterized by normal macrocirculatory parameters, while microcirculatory function is impaired. However, direct assessment of the microcirculation in clinical practice is largely limited to indirect markers.

Direct visualization of the microvascular bed is feasible using hand-held vital microscopy (HVM) technologies, including sidestream dark-field (SDF) and incident dark-field (IDF) imaging. These devices allow real-time, noninvasive imaging of microcirculation directly at the patient’s bedside. In neonates, commonly used imaging sites include the sublingual and buccal mucosa, as well as transcutaneous visualization of the upper inner arm and axilla [82]. However, the use of HVM devices is still largely experimental and microcirculatory monitoring has yet to be incorporated into standard clinical care.

Since its introduction, HVM has been researched in neonates, mainly in small-scale observational studies. A major limitation remains the absence of standardized reference values for microcirculatory parameters in neonates and infants [82].

One small pediatric study found that patients with cyanotic CHD show higher vessel densities than those with acyanotic CHD [83]. A recent single-center study investigated the microcirculation of patients with heart disease, before and after cardiac surgery [84]. Compared to children without CHD, cardiac patients showed similar perfused vessel densities and red blood cell velocities before surgery, but less perfused vessels, lower perfusion quality, and higher small vessel densities than children without CHD. After surgery, perfused vessel densities and perfusion quality of small vessels declined, while red blood cell velocities increased.

Nussbaum et al. employed SDF imaging to study the effects of cardiopulmonary bypass on skin microcirculation, reporting transient disturbances in glycocalyx thickness, microvascular flow index (MFI), and perfused vessel density (PVD), which normalized within a week postoperatively [85]. Scolletta et al. evaluated sublingual microcirculation in infants with cyanotic and acyanotic CHD using SDF, noting that while acyanotic infants maintained stable microvascular parameters, those with cyanotic CHD showed significant reductions in total vessel density (TVD) and proportion of perfused vessels (PPV) [86].

## 5. Future Directions

Advancements in biomedical technology and computer science have increased the capabilities of monitoring systems to collect, store, and analyze complex data. The development of a hemodynamic monitoring “tower” [87] facilitates continuous, real-time data acquisition at the bedside by incorporating data from multiple monitoring devices simultaneously. This allows the trend analysis of fluctuating hemodynamic parameters and the relationship between them.

**Figure 8:**
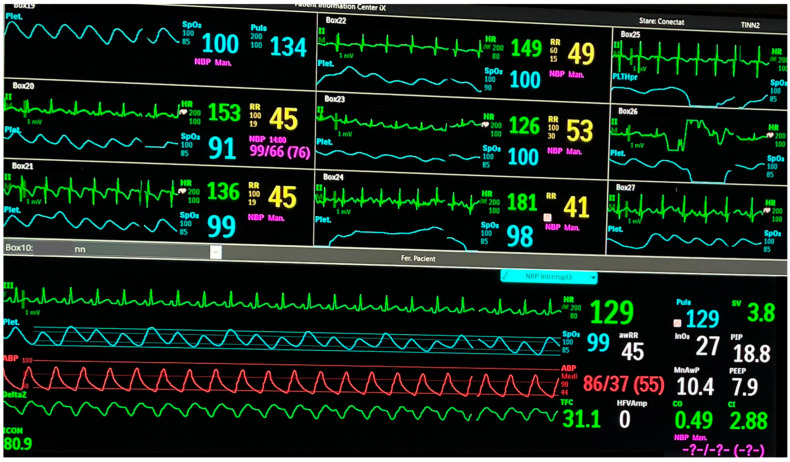
The tele-ICU room in the Neonatal Intensive Care Unit at the “Maria Sklodowska Curie” Emergency Clinical Hospital for Children with central monitoring system spread out over an entire wall, with detailed vital signs and high-resolution cameras for all patients.

**Figure 9:**
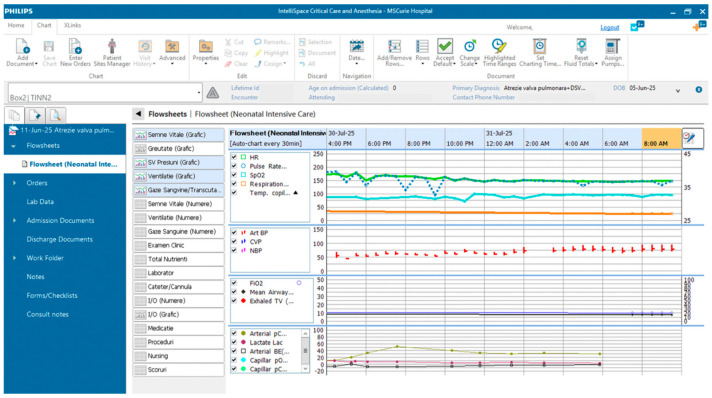
ICCA (IntelliSpace Critical Care and Anesthesia) system in the Neonatal Intensive Care Unit at the “Maria Sklodowska Curie” Emergency Clinical Hospital for Children, which centralizes all the data coming from the medical equipment and shows graphic and numeric representation of vital signs, ventilator settings.

The implementation in the clinical setting presents considerable challenges and requires substantial financial investment and digital infrastructure capable of storing the ever-growing volume of patient data. At the Neonatal Intensive Care Unit at the “Maria Sklodowska Curie” Emergency Clinical Hospital for Children, a multimodal monitoring platform is currently operational (see Figure 8 and Figure 9). This system integrates real-time data streams from bedside monitors (including heart rate, blood pressure, respiratory rate, SpO_2_, and transcutaneous CO_2_), near-infrared spectroscopy (cerebral and renal SO_2_), ventilator settings, ICON parameters, amplitude-integrated EEG (aEEG), and echocardiographic findings. Beyond clinical utility, this platform serves as a research tool. Nevertheless, a significant challenge lies in the accurate and synchronized manual documentation of therapeutic interventions, particularly regarding medication dosages and infusion timings, alongside hemodynamic and respiratory metrics. The lack of full integration between infusion pumps and the central informatics infrastructure remains a significant limitation.

A multimodal monitoring system enables the real-time acquisition of heterogeneous data from multiple devices, each characterized by varying degrees of accuracy across different clinical scenarios and subject to intrinsic limitations. The simultaneous integration and analysis of these diverse data streams provide a more comprehensive characterization of the patient’s status, with data from each monitoring system complementing one another like pieces of a puzzle. Consequently, clinical decisions can be better informed and tailored to the specific characteristics of the patient and their pathology, thereby facilitating personalized management.

## 6. Conclusions

Clinical assessment of hemodynamics using indirect markers of cardiac output and systemic vascular resistance has demonstrated limited accuracy. The ideal monitoring method for the detection of subtle hemodynamic changes following cardiac surgery should be noninvasive, safe, accurate, cost-effective, easy to use at the patient’s bedside, designed for pediatric patients, and capable of accounting for intra- and extracardiac shunts. Furthermore, it should enable continuous, automated data recording without requiring constant manual input. There is currently no single device that meets all these requirements; consequently, each intensive care unit chooses its own monitoring strategy in relation to equipment availability, patient characteristics, clinical needs, and physician experience.

The most recently developed devices for hemodynamic monitoring are not yet routinely implemented in clinical practice, and current expert recommendations support their use primarily within research settings. Among the most promising technologies for continuous noninvasive monitoring are electrical biosensing technologies and hand-held devices for evaluating the microcirculation. Key challenges include difficulties in validation against established reference methods, lack of measurement standardization, no consensus for guiding treatment, as well as limited data regarding their impact on patient outcome. These newer technologies can only be used as part of a multimodal hemodynamic monitoring, alongside intermittent methods such as echocardiography and regional perfusion assessment tools, including near-infrared spectroscopy (NIRS). A comprehensive strategy combining global and regional hemodynamic data is necessary for guiding individualized, pathophysiology-based therapeutic strategies. Further studies are expected to show the impact of this global, multimodal hemodynamic monitoring strategy on the short-term and long-term outcomes in neonates with congenital heart disease.

## Figures and Tables

**Table 1:** The main scores used for hemodynamic monitoring in the Post-Cardiac Neonatal Intensive Care Units.

	Low Cardiac Output Syndrome Score (LCOSS) [30,33]	Vasoactive Inotropic Score (VIS) [48]	Ventilation Index (VI)	Vasoactive-Ventilation-Renal Score (VVR) [47]
Parameters	heart rate (HR) urine output (UO) lactate level peripheral temperature * fluid bolus requirement vasoactive-inotrope requirement ** cerebral and renal oxygen saturation (ScO_2_) ***	dopamine dose (μg/kg/min) dobutamine dose (μg/kg/min) epinephrine dose (μg/kg/min) norepinephrine dose (μg/kg/min) milrinone dose (μg/kg/min) terlipressin dose (μg/min) vasopressin dose (U/kg/min) levosimendan dose (μg/kg/min) methylene blue dose (mg/kg/h) phenylephrine dose (μg/kg/min) enoximone dose (μg/kg/min) olprinone dose (μg/kg/min) angiotensin II dose (ng/kg/min)	ventilator respiratory rate (RR) peak inspiratory pressure (PIP) positive end-expiratory pressure (PEEP) arterial carbon dioxide pressure (PaCO_2_)	ventilation index (VI) vasoactive inotropic score (VIS) ΔCreatinine ****
Formula	Assign 1 point for: HR > 20% for age-adjusted normal value UO < 1 mL/kg/h Lactate > 2 mmol/L Toe temperature < 30 °C OR CRT > 3 s * Fluid bolus requirement > 30 mL/kg/day Vasoactive-inotrope requirement > milrinone 0.5 μg/kg/min OR VIS > 5 ** Cerebral NIRS < 50% and renal NIRS < 75% ***	10,000 × vasopressin dose + 100 × epinephrine dose + 100 × norepinephrine dose + 50 × levosimendan dose + 25 × olprinone dose + 20 × methylene blue dose + 10 × milrinone dose + 10 × phenylephrine dose + 10 × terlipressin dose + 0.25 × angiotensin II dose dobutamine dose + dopamine dose + enoximone dose	(RR × [PIP − PEEP] × PaCO_2_)/1000	VI + VIS + (ΔCreatinine × 10)

* Decreased peripheral temperature is defined as a toe temperature < 30 °C according to Ulate et al. [30] and as a capillary refill time > 3 s according to Aslan et al. [33]. ** Increased vasoactive–inotrope requirement is defined as exceeding milrinone 0.5 μg/kg/min according to Ulate et al. and as a vasoactive–inotrope score > 5 according to Aslan et al. *** Both Ulate et al. and Aslan et al. define decreased cerebral oxygenation as ScO_2_ < 50% measured by near-infrared spectroscopy (NIRS), while Aslan et al. also define decreased renal oxygenation < 75% measured by NIRS. **** ΔCreatinine represents the change in creatinine from baseline.

## Data Availability

Not applicable.

## Appendix A

**Table A1:** Literature search strategy.

PubMed	Scopus
((“neonate”[All Fields] OR “newborn”[All Fields] OR “infant”[All Fields]) AND (“congenital heart disease”[All Fields] OR “heart disease”[All Fields] OR “CHD”[All Fields]) AND (“hemodynamic”[All Fields] OR “hemodynamics”[All Fields]) AND (“monitoring”[All Fields] OR “monitor”[All Fields]))	TITLE-ABS-KEY ((((neonate OR newborn OR infant) AND (“congenital heart disease” OR “heart disease” OR CHD) AND (hemodynamic OR hemodynamics) AND monitoring OR monitor))))

**Table A2:** Results of the literature search up to July 2025.

Database	References	References After Title and Abstract Screening
PubMed	56	29
Scopus	189	53
Manual search	13	13
Total articles	167	75 (after duplicates’ removal and adjunct of articles from manual search)
